# Young transgender individuals' lived experiences of facing life's challenges: a qualitative study in Iran

**DOI:** 10.3389/fpubh.2023.1134237

**Published:** 2023-06-15

**Authors:** Fateme Mohammadi, Seyedeh Zahra Masoumi, Banafsheh Tehranineshat, Khodayar Oshvandi, Mostafa Bijani

**Affiliations:** ^1^Department of Nursing, Chronic Diseases (Home Care) Research Center and Autism Spectrum Disorders Research Center, Hamadan University of Medical Sciences, Hamadan, Iran; ^2^Department of Midwifery, School of Nursing and Midwifery, Mother and Child Care Research Center, Hamadan University of Medical Sciences, Hamadan, Iran; ^3^Department of Nursing, Faculty of Nursing and Midwifery, Hormozgan University of Medical Sciences, Bandar Abbas, Iran; ^4^Department of Mother and Child Care Research Center, Hamadan University of Medical Sciences, Hamadan, Iran; ^5^Department of Medical Surgical Nursing, School of Nursing, Fasa University of Medical Sciences, Fasa, Iran

**Keywords:** transgender, qualitative study, social support, life experiences, life challenges

## Abstract

**Introduction:**

In recent years, several studies have addressed the challenges and psychological issues that transgender individuals face. However, only a few studies have explored the experiences of this population in Iran. Life experiences are heavily influenced by the dominant religious and cultural conditions and common beliefs in a society. The present study aimed to investigate transgender individuals' lived experiences of facing life's challenges in Iran.

**Methods:**

This is a qualitative study with a descriptive and phenomenological design conducted from February to April 2022. Data were collected using semi-structured, in-depth interviews with 23 transgender individuals (13 AFAB and 10 AMAB). The collected data were analyzed using Colaizzi's method.

**Results:**

Analysis of the qualitative data resulted in the emergence of three themes and 11 subthemes. The three main themes included mental health disparities (fear of having one's secret revealed, depression and despair, suicidal thoughts, and secrecy in the family), gender dysphoria (contradictory gender identity and contradictory behaviors), and stigma and insecurity (sexual abuse, social discrimination, disrupted occupational performance, a lack of support, disrepute, and disgrace).

**Conclusions:**

The findings of the study indicated that transgender people in Iran are exposed to considerable mental health disparities. In addition to disrepute, infamy, and stigma, transgender people face sexual abuse, social discrimination, and a lack of family social support. The results of the present study can help mental health experts and the healthcare system as a whole to adjust their mental and physical health programs according to the needs and experiences of transgender people and their families. It is recommended that future research address the problems and psychological challenges that transgender people's families have to confront.

## Introduction

One of the most important aspects of an individual's human identity is their gender identity, which forms in the process of socialization in formal and informal institutions (1, 2). Gender identity represents an individual's perception of their femininity or masculinity (2). Moreover, the ideas and attitudes that are defined regarding appropriate male and female behaviors in the culture of any society are effective in the formation of gender identity (1, 2). The gender binary describes the inaccurate concept that gender classifies human beings into only two distinct forms (i.e., man and woman). People who are assigned male at birth (AMAB) are expected to identify as boys and men and exhibit masculinity, and people who are assigned female at birth (AFAB) are expected to identify as girls and women and exhibit femininity (3).

Occasionally, an individual may have the biological characteristics of a male or female but not have a mental sense of belonging to that gender. Such an individual is called a transgender person (2). In the Diagnostic and Statistical Manual of Mental Disorders, 5th edition, this condition is known as gender dysphoria (4). It is estimated that, globally, the prevalence of feminine behaviors among males is one in 30,000, and the inclination toward masculine behaviors among females is one in 100,000 (5). There is a lack of accurate statistics on transgender people in Iran, but it is estimated that ~4,000 transgender people are currently living in this country (6). In recent years, with an increase in transgender people in the world, some studies have investigated the problems and challenges faced by these people, and these studies have been conducted in different social and cultural contexts in Iran (7, 8). However, only a few studies have explored the experiences of this population in Iran. In this regard, Afrasiabi and Junbakhsh (9) stated that these individuals attempt to save their desired identity through daydreaming and performing identity roles in solitude. Moreover, Eftekhar et al. (10) postulated that transgender women would use sexual practices to be approved/accepted as women, and the use of sex for this purpose led them to engage in high-risk sexual relationships.

The study of Nematollahi et al. showed that transgender women are at risk of depression, anxiety, and stress. These conditions are closely related to their quality of life. Moreover, transgender women suffer from high levels of sexual violence that affect their mental health. There is a strong requirement to promote the culture of preventing violence against women in Iranian society (11). Another study in Iran showed that transgender individuals suffer from mental health disorders before surgery. In addition, gender-reassignment surgery and job creation are the most effective strategies for improving body image and quality of life among individuals with gender identity disorders (12–14).

These studies indicated that more extensive studies should be conducted in Iran in this regard since sexual matters are sensitive in the Iranian culture and can pose a threat to an individual's dignity or the dignity of their family. Moreover, life experiences are deeply influenced by religious and cultural beliefs. Transgender people suffer from discrimination and face life challenges. Life challenges are problems and issues that make people's lives difficult and interfere with their ability to achieve their life goals. These psychosocial issues are beyond their control and deeply affect transgender people's lives. Thus, the present study aimed to investigate transgender individuals' lived experiences of facing life's challenges in Iran.

## Methods

### Study design and research question

This is a qualitative study with a descriptive and phenomenological design. The study was conducted from February to April 2022. Descriptive phenomenology is a philosophy and a scientific approach that investigates and describes individuals' lived experiences (15). The goal of the phenomenological approach was to clarify specific phenomena according to how they are perceived by the individuals involved. In the humanity domain, this is equivalent to collecting in-depth information and perceptions through inductive, qualitative methods, including interviews, discussions, and participant observation, and showing them from the participants' perspective (16). Phenomenology deals with the study of experiences from the viewpoint of a certain individual, leaving out taken-for-granted beliefs and usual ways of understanding things. This type of design is appropriate when the existing knowledge or research literature on a phenomenon is inadequate. Researchers avoided using preconceived categories but they allowed categories and names for categories to originate from the data. Researchers immersed themselves in the data to achieve new insights (17). Hence, according to a few studies on the experiences of transgender individuals in Iran, given the sensitive nature of this topic and how culture and religion strongly influence the lives of people in Iran, the present study was conducted with a descriptive phenomenological design, which uses a qualitative approach to examine the experiences of this population in Iran, which have never been studied before in Iran.

### Participants

The participants comprised 23 transgender individuals with a wide range of demographic characteristics regarding age, gender, work experience, and so on. Creswell and Creswell (18) claimed that the sample size for conducting qualitative research is usually 20–30 participants.

The inclusion criteria were as follows: identification as transgender, verification through medical records and diagnosis by the attending physician, willingness to participate in the study, Iranian nationality, proficiency in Farsi, and ability to effectively express and provide comprehensive and appropriate information. The first author contacted two welfare centers affiliated with a university of medical sciences in the west of Iran and acquired the phone numbers of all the transgender individuals registered there. Then, the corresponding author contacted these individuals, explained the study's objectives to them, and asked them to participate. In this study, 43 transgender people were introduced, but only 23 of them were willing to participate in the study and were willing to be interviewed. For participating in the study, each participant was given $10. The participants were selected via purposeful sampling. Purposeful sampling is a method used in a qualitative study to identify and select individuals or groups of individuals who are especially knowledgeable about or experienced in a particular phenomenon (19). Moreover, interviews continued until the data were saturated. Saturation occurs when no new categories emerge, and the categories are saturated based on their characteristics and dimensions.

### Data collection

Data were collected from 23 individual, semi-structured interviews. Four key questions were initially selected based on a review of the literature. To develop and test the questions, two interviews were conducted in the presence of all the research team members. Based on the outcome, an interview plan was developed. The participants were interviewed via video calls on WhatsApp, which were scheduled at mutually convenient times. The corresponding author conducted and analyzed all the interviews. Each interview began with a few general questions, including “Can you describe a typical day of your life?”, “What feelings have you experienced?”, “How has being a transgender person affected your life?”, and “How have you lived with this challenge so far?”. Subsequently, based on the respondents' initial answers, follow-up questions were asked to add to the clarity of the information; these questions included, “Can you explain further?”, “What do you mean by that?”, and “Can you give an example?”. The interviews centered on the main objective of the study. Each interview lasted between 30 and 40 min. Upon interview completion, each interview was repeatedly listened to by the first author and the corresponding author several times to develop a general understanding and deep insights.

Then, the interviews were transcribed. After extracting the semantic units and classifying these semantic units for each interview, the two authors conducted a meeting in the room of the corresponding author and agreed on the semantic units, the titles assigned to the semantic units, and the classification and naming of the categories. The interview continued until the data were saturated and no new categories could be extracted. The data were organized using the MAXQDA software program. The collected data were analyzed using the Colaizzi's method, which consists of seven steps: (1) reading and re-reading every transcribed interview, (2) extracting important terms and phrases from the transcripts, (3) assigning meaning to the extracted units, (4) organizing and categorizing similar units, (5) providing comprehensive descriptions of the extracted categories, (6) creating a basic paradigm of the subject under study according to the extracted categories, and (7) confirming the basic paradigm by having the participants verify the themes and categories (20).

### Rigor

Guba and Lincoln's criteria were used to evaluate the trustworthiness of the study (21). To enhance the credibility and accuracy of the data, the researchers used a mixed-methods approach: semi-structured interviews, prolonged engagement with the data, member checking, and peer checking. The extracted concepts and themes were shown to two of the participants and three peers who were experts in qualitative studies and who interacted with transgender people (a psychologist, a psychiatrist, and a psychiatric nurse). They confirmed that the findings were consistent with their understanding and interpretations. In addition, the researchers limited the textual reviews to reduce bias in conducting, analyzing, and coding the interviews and to enhance the validity of the data. Finally, confirmability was increased through accurate recording of the participants' narratives and detailed reporting of the study results to make it possible for other researchers to follow up on the findings.

### Ethical considerations

The present study was conducted using the revised Declaration of Helsinki, a statement of ethical principles that directs physicians and other participants in medical research involving human subjects. All participants provided informed consent to participate in the study. The participants were assured of the anonymity and confidentiality of their information. Moreover, the local Ethics Committee approved the study at Hamadan University of Medical Sciences in the west of Iran (Ethical code: IR.UMSHA.REC.1401.477).

## Results

The participants consisted of 13 AFAB and 10 AMAB. The average age of the participants was 32.04±2.54 years. A total of eight participants (four AFAB and four AMAB) had started treatment under the supervision of a specialist doctor, two were candidates for surgery, and six were undergoing hormone therapy. According to the interviews with transgender people, the beginning of the treatment made them feel calm and reduced their depression, but they still faced the challenge of family and society's acceptance. The majority of them (86.95%) had a high school diploma. Additionally, most of them (91.30%) had an income of approximately $US 70 during the last 12 months. In addition, most of them (86.95%) lived independently and far from their families (Table 1).

**Table 1:** Individual social characteristics of the participants.

**Participants**	**Sex**	**Age (year)**	**Job**	**Educational level**
P1	AFAB	32	Self-employed	High-school diploma
P2	AMAB	40	Self-employed	Bachelor
P3	AFAB	41	Self-employed	High-school diploma
P4	AMAB	27	Farmer	High-school diploma
P5	AFAB	34	Rancher	High-school diploma
P6	AMAB	38	Farmer	High-school diploma
P7	AFAB	33	Employee	Bachelor's degree
P8	AMAB	38	Self-employed	Bachelor's degree
P9	AFAB	39	Self-employed	High-school diploma
P10	AMAB	34	Self-employed	High-school diploma
P11	AMAB	30	Farmer	High-school diploma
P12	AFAB	37	Self-employed	High-school diploma
P13	AFAB	28	Self-employed	High-school diploma
P14	AFAB	24	Self-employed	High-school diploma
P15	AFAB	28	Employee	Bachelor's degree
P16	AMAB	32	Rancher	Bachelor's degree
P17	AMAB	22	Rancher	High-school diploma
P18	AFAB	26	Self-employed	High-school diploma
P19	AMAB	24	Rancher	High-school diploma
P20	AFAB	27	Self-employed	High-school diploma
P21	AMAB	32	Rancher	High-school diploma
P22	AFAB	29	Self-employed	High-school diploma
P23	AFAB	42	Rancher	Bachelor's degree

Analysis of the qualitative data resulted in the emergence of three themes and 11 subthemes. The three main themes were mental health disparities, gender dysphoria, and stigma and insecurity.

### Mental health disparities

In total, 18 transgender people reported mental health disparities such as the fear of having their sexual identity revealed, non-acceptance by society, and a lack of emotional support, which severely affected their mental health. The theme of mental health disparities consisted of the fear of having one's secret revealed, depression and despair, suicidal thoughts, and secrecy in the family.

#### Fear of having one's secret revealed

Most participants reported that they feared their secret would be revealed and everyone would find out that they were transgender. People in this population have considerable fear and anxiety. They have to deal with the fear of being found out every day and going through serious psycho-emotional crises. One of the participants stated the following:

*My whole life is filled with fear and anxiety about what I should do if people find out about my gender dysphoria. I'm really worried that my friends and relatives might find out that I'm transgender people. Some of my close relatives have become suspicious of my physical appearance and unusual behaviors, but they don't know about my gender dysphoria yet. But, how long can I keep this a secret? They're going to find out sooner or later; the truth will (sic) out some day* (Participant 6).

#### Depression and despair

All of the participants reported that transgender individuals experience high degrees of depression and despair, which could lead them to lose hope in life, isolate themselves, and avoid social contact. One of the participants stated the following sentiments:

*I'm really tired. My life feels meaningless. How long should I take antidepressant pills? They don't do me any good and every day I'm feeling worse. Would you call this living? How long do I have to live like this and be afraid of my own shadow?* (Participant 5).

#### Suicidal thoughts

Most of the participants said that the non-acceptance of transgender people by their families and society, as well as the negative views and inappropriate behavior of people around them and sometimes even inappropriate words and stigma surrounding them, causes severe depression and promotes suicidal behaviors. One of the participants said this:

*We haven't done anything wrong. We just have a problem and something must be done about it. Some of my friends who were transgender people became drug addicts or attempted suicide and died since their families and society did not support them and they felt rejected. Unfortunately, not many awareness campaigns have been set up to increase people's awareness of transgender people, and many see us as perverts and sexual deviants or social deviants. This attitude is (sic) a torture worse than death* (Participant 9).

Another participant expressed the following:

*I've wanted to commit suicide many times and free myself of all the disrespect and pain that I have to bear from people's unkind looks. How much am I supposed to tolerate this?* (Participant 7).

#### Secrecy in the family

Another subtheme of mental health disparities was secrecy in the family. A total of 17 participants said that families hide their children's sexual desires from relatives and society. This subject caused psycho-emotional tension, anxiety, frustration, and diminished psychological security in transgender people and families, adversely affecting all aspects of their social lives. One of the participants stated the following:

*I've had a lot of arguments with my parents over why I should keep my sexual desires a secret; I've been scolded and even insulted. But how long can I hide this and lie to everyone? What have we done to have to hide our disorder?* (Participant 3).

### Gender dysphoria

Another theme extracted from the collected data was gender dysphoria. In total, 19 participants said that gender dysphoria was an inevitable crisis that affected all personal and social aspects of their lives. The theme of gender dysphoria is comprised of contradictory gender identities and contradictory behaviors.

#### Contradictory gender identity

Most of the participants said that they do not like the gender that they were born with, and they tend to have the identity of the opposite sex, which is not accepted in Iranian society, since they experience contradictory gender identities. One of the participants said the following:

*I don't like the female gender; I have strong male tendencies. I've wanted to undergo surgery, leave this feminine body and look like a man several times. My family won't allow it. They say if I do it, they will disown me* (Participant 10).

#### Contradictory behaviors

The participants reported experiencing contradictory behaviors and interactions with their assigned gender at birth. A lack of compatibility between their gender identity and their assigned male or female body, engaging in activities such as wearing make-up and feminine clothing, experiencing hatred toward their genitals and female body parts, as well as adopting male clothing and behaviors were examples of contradictory behaviors associated with gender dysphoria.

One of the participants stated that,

*Many times contradiction in my gender-related behaviors in my social and personal interactions has caused me mental and emotional shock. For example, one day, when I went shopping at a store, the other customers mocked me. One of them said, “Strange! Why does this guy look like a woman?' another said, “I don't think she was a woman. He seemed more like a man.”*(Participant 16).

### Stigma and insecurity

Another theme extracted from the data in the present study was stigma and insecurity, as experienced by 20 participants. This theme comprises sexual abuse, social discrimination, disrupted occupational performance, a lack of support, and disrepute and disgrace.

#### Sexual abuse

One of the psycho-emotional issues that threaten the security of transgender individuals is sexual abuse, as reported by 15 participants, nine AFAB and six AMAB. Moreover, this type of abuse is more prevalent in childhood and adolescence. One of the participants stated the following:

*A friend of mine who is transgender people once told me that when he was serving in the military, he was sexually assaulted and abused by some other soldiers who were his roommates* (Participant 17).

#### Social discrimination

Most of the participants revealed that one of the greatest challenges in their lives was being rejected by society and experiencing social discrimination. The absence of social justice and lack of access to cultural/social/recreational/educational facilities were the major examples of social discrimination against them. According to one of the participants,

*It's like we are not part of this society. Because of our disorder, they deprive us of our right to welfare and social services. What's the difference between us and other people? Like everyone else in the society [sic], we are entitled to have social rights as citizens* (Participant 15).

#### Disrupted occupational performance

All participants stated that they were experiencing considerable occupational stress and that their gender dysphoria threatened their job security. One of the participants expressed the following sentiments:

*Our job prospects are really unclear. When we are not accepted by the society [sic], how can we have job security? For instance, I passed the employment test of a bank, but when they found out about my gender dysphoria [transgender people], they refused to employ me. My other friends that I'm in touch with have the same problem: none of them has job security or can find a job at all* (Participant 18).

#### Lack of support

Lack of support was another subtheme of stigma and insecurity in the present study. Based on 17 participants' experiences and views, individuals with gender dysphoria are in urgent need of a family and social support system. Lack of support threatens their security and aggravates their psycho-emotional crises and personal, family, and social stress. One of the participants stated the following:

*Most of the time, I feel really lonely. I don't think anyone cares about me, and I feel worthless and frustrated. I don't know where to seek refuge or who to ask for help. I only have hope in God's help, and it's only this belief in God that's making it possible for me to continue my life* (Participant 19).

#### Disrepute and disgrace

As another subtheme of disrepute and disgrace, disrepute and disgrace caused great concern to 17 of the participants and affected all personal and social aspects of their lives. One of the participants stated the following concerns:

*Fear of stigma and a bad reputation is eating me. I feel ashamed. I feel I've lost my social and human dignity, don't have any value in the society [sic], and have brought shame to my family* (Participant 14).

## Discussion

The findings of the present study indicated that the lived experiences of young transgender individuals could be categorized into three main themes: mental health disparities, gender dysphoria, and stigma and insecurity.

The young transgender people in the present study experienced serious psycho-emotional issues that influenced their psychological security as well as their personal lives and social activities. The theme of mental health disparities consisted of the fear of having one's secret revealed, depression and despair, suicidal thoughts, and secrecy in the family. In a study by Kade (22), the revelation of the identity of male transgender people was affected by a number of underlying factors, including accurate gender determination, discrimination, stigmatization, emotional intimacy, and awareness of physical changes. While male transgender people were inclined to reveal their gender to others, they avoided contact with others for fear of potential violence, discrimination, and stigmatization (22). According to several studies, transgender individuals' physical and mental health is unsatisfactory, and they are at higher risk of anxiety, depression, drug abuse, and suicide than the general population (23–27). In one study 2016, transgender adolescents complained of being insulted and humiliated (24). In a study by Guss et al. (28), adolescent transgender people had unpleasant experiences of losing hope and being rejected by healthcare providers and strongly demanded respect for their gender identity and for maintaining confidentiality. Most of the transsexual participants in the study of Testa et al. (29) had experienced physical violence and suicidal thoughts. Women with transgender children were ashamed of their offspring, especially in the beginning when they realized their sexual orientation (23).

Moreover, Nematollahi et al. (11) stated that these patients experience a lot of tension and depression in the cultural context of Iran, which has negative effects on their mental health. Many studies have reported high rates of mental health issues, including depression, anxiety, suicidal thoughts, or suicide attempts, in individuals who belong to a sexual minority (30). Meyer's minority stress model posits that sexual minorities face unique and hostile stressors (e.g., homophobic victimization) related to their sexual minority identity. Consequently, these stressors have negative effects on their health. Moreover, sexual minorities are exposed to many negative health outcomes, including prejudgment, stigma, discrimination, rejection, insult, and other forms of aggression (31). Minority stress refers to severe chronic anxiety experienced by the members of a minority in response to being pressured, despised, and labeled (32). In this model, the social environment's conditions are considered stressors. Minority stress encompasses proximal stressors (stressors on the personal/individual level, such as internalized stigma) and distal stressors (stressors on the social level, such as discrimination) (33, 34). As with previous research, the findings of the present study indicated that, in Iranian culture, negative attitudes toward transgender people and a lack of effective cultural and social awareness campaigns have caused many of these individuals to suffer from various stressors and mental disorders, including depression and anxiety, as well as a lack of support from their families and society. To avoid these consequences, there is an urgent need for psychological therapies (individual, group, and family interventions) and for educating the public about these minorities.

Gender dysphoria was another theme extracted from the data in the present study. The gender dysphoria of the participants influenced all personal and social aspects of their lives. The theme of gender dysphoria consists of contradictory gender identities and contradictory behaviors. Afrasiabi et al. (9) stated that transgender youth in Iran report that there are incompatibilities between their bodies and minds; therefore, they experience contradictory gender identities that make them susceptible to mental disorders, including depression, hostility, and phobia. According to a review study by Lin et al. (33), transgender people like to live and behave according to their chosen identity. However, these behaviors may be different from their birth identity. These behavioral differences cause people in society to not interact properly with them and are not accepted (33). Yang et al. (35) stated that the behavior that contradicts the gender identity of transgender people at birth causes people in society to look at them with strange looks, avoid them, and not establish proper interaction with them, which is in line with the present study. In a study, counseling transgender people on feeling less ashamed of their identity, self-disclosure, and expressing positive identity experiences was found to be useful (36). In Western cultures, public opinion of transgender people differs greatly from that of the Iranian culture. In the west, with the help of social support groups, families attempted to provide their transsexual children with comprehensive support, and they focused on their children rather than the attitudes and reactions of society. In Iran, however, what matters most to the families of transsexual individuals is public opinion and protecting their reputation (8). Raising awareness among mental health professionals via the media and public organizations is essential. Further, positive clinical environments and family therapy for the families of transsexual people can prove to be helpful. Healthcare professionals should be educated about gender transition and transgender people's culture and cultural values.

Stigma and insecurity was another category of the experiences of young transgender people in the present study. This theme comprised sexual abuse, social discrimination, disrupted occupational performance, a lack of support, and disrepute and disgrace. In many studies involving transgender individuals, the study population mentioned being stigmatized in educational environments, workplaces, and other social venues, and many of them deemed it necessary to hide their gender identity. In a study by Bowling et al. (23), one of the negative consequences of being transgender was a social stigma, leading to loneliness, isolation, hiding one's identity, and depression. In their study, Yang et al. (35) found that experiencing stigma led to mental health disorders, including depression and severe anxiety. Trans women experienced various forms of stigma, maltreatment, disrespect, and inequality in accessing decent employment and dwelling places, as well as healthcare and legal services (35). Plöderl et al. (31) stated that transgender people's experiences of healthcare services are replete with feelings of humiliation and deprivation. Despite their special needs, they are not understood by professional healthcare providers. As a result, they cannot speak openly about their identity and feel doubtful or ashamed (31). Other studies have highlighted the social crisis and stigma resulting from inadequate awareness of transgender individuals, particularly concerning the refusal of school authorities and parents of other students to accept them.

To avoid the negative attitude and criticism of their society, the families of some transgender people moved to other countries or larger cities or even made their children immigrate alone (37, 38). In a study by Fernandez-Rouco (37), the transsexual subjects had been victims of sexual violence and child abuse, which disrupted their close relationships and caused feelings of shame and guilt, anxiety, and anger among the victims. Eftekhar et al. (10) said that transgender people in Iran engage in risky sexual relations, which causes them to become infected with AIDS and hepatitis. Katz-wise et al. (39) reported uncertainty about the future, concerns about their physical and emotional security, and the problems faced by young transgender people. Transgender people's parents were highly concerned about their children's romantic relationships in the future. Social factors such as discrimination in employment and access to social services give transgender people a negative view of the future. Transgender people's parents were also concerned about the lack of physical and emotional security of their children against sexual violence and abuse, as well as society's refusal to accept transgender people's identities (39). In their study, Seibel et al. (40) concluded that transgender people's failure to be accepted and supported by their families causes them to flee from home, face homelessness, and lose their self-esteem. However, another study reported that transgender individuals who maintain close relationships with their families and receive support from them experience higher levels of self-esteem, develop a positive self-image, gain access to a stronger support system, and perceive greater social support (41). Several studies have reported that a lack of support for transgender people causes them to flee from home, engage in inappropriate behaviors and drug abuse, and experience a lack of family support (42–44). In a study by Zeluf et al. (45), the transsexual participants had experienced limited access to social services and a lack of social support, and as such, they had a low quality of life. In a study, many transgender people avoided social contact due to feelings of fear and insecurity. However, engaging in online communication within transsexual communities on social networks emerged as a positive factor, enhancing their emotional health and fostering resilience (46). According to several studies, one of the greatest concerns for transgender people is employment. Because of their appearances or special gender identities, many of these individuals cannot find jobs that are compatible with their academic fields. Thus, they face problems such as fear of their gender identity being revealed at work, feeling frustrated and incompetent, and a lack of job security and stability. They have to resort to any means since they do not have financial sponsors. At work, transgender individuals are called names, stigmatized, and sexually abused (46, 47). Group therapy sessions for the families of individuals with gender identity disorders can help family members interact with each other, normalize the situation, and become involved in treatment. Transgender people should be encouraged to communicate with each other through online social networks and in person. In addition, societies and organizations should be established to address the issues of individuals with gender identity disorders and encourage them to participate in group activities to reduce their social isolation. Awareness campaigns can help modify wrong and inappropriate perceptions of transgender people and promote more realistic attitudes. Educating families and society and developing educational programs for the media can replace the dominant attitude toward transgender people with more humane and moderate perceptions of this population, which will, in turn, alleviate their problems.

## Limitations

One limitation of the present study was the small size of its study population, which may not represent all transgender people. Moreover, there is a lack of generalization of the findings due to the probability sampling method. Moreover, the lived experiences of transgender people who were not willing to speak to the researcher were not included, which may limit the transferability of the results. It is recommended that more studies need to be conducted to investigate the experiences of this population in different cultural contexts. Another limitation of the study was that, because of the COVID-19 pandemic, the participants were not interviewed face to face. However, the researchers used video calls to improve their interaction with the participants.

## Conclusion

The findings of the study suggest that participants in Iran are subject to considerable mental pressure. In addition to disrepute, infamy, and stigma, participants are faced with sexual abuse, social discrimination, unemployment, and a lack of family and social support. Moreover, concerns about the future, despair, and suicidal thoughts are other issues these people in Iran have to cope with. Raising public awareness, avoiding bias against transgender people, and not criticizing them or their families are essential to preserving their dignity. The results of the present study can help mental health experts and the healthcare system as a whole to adapt their mental and physical health programs according to the needs and experiences of these people and their families. It is recommended that further studies need to be conducted to investigate the experiences of this population across different cultural contexts.

## Data availability statement

The original contributions presented in the study are included in the article/supplementary material, further inquiries can be directed to the corresponding author.

## Ethics statement

In the present study, individuals who met the inclusion criteria of the study were identified after the researchers had obtained permission from the Ethics Committee of the research department at the University of Medical Sciences in the west of Iran (IR.UMSHA.REC.1401.477). The patients/participants provided their written informed consent to participate in this study.

## Author contributions

FM, MB, and SZM were involved in the conception and organization of the study. SZM, FM, and KO were involved in the execution and data collection of the study. FM and BT participated in statistical analysis design and/or execution. All authors contributed to the preparation and critical review and approved the final version of the manuscript.
